# Wireless trigger distribution with nanosecond jitter based on ultra-wideband transceiver modules

**DOI:** 10.1016/j.ohx.2025.e00713

**Published:** 2025-10-08

**Authors:** Julius Korsimaa, Martin Weber, Edward Hæggström, Ari Salmi

**Affiliations:** Electronics Research Laboratory, Department of Physics, University of Helsinki, P.O.B. 64, FI-00014 University of Helsinki, Finland

**Keywords:** Triggering, Synchronization, Ultra-wideband, Ranging, Internet of Things (IoT), Sensor network, Industry 4.0

## Abstract

Wireless sensor networks require time synchronization to operate in a coordinated manner. Synchronizing the clock of each sensor node by using a network protocol can be sufficient for millisecond accuracy, whereas precision in the range of nanoseconds can be achieved by using Global Navigation Satellite System (GNSS) receivers.

Unfortunately, the use of GNSS signals requires an unobstructed view of the sky, and thus they cannot be used indoors or underground. Additionally, GNSS is susceptible to jamming and spoofing, and their use depends on the availability of global infrastructure.

To address these limitations, we propose a trigger signal distribution system based on the DWM1000 IEEE 802.15.4-2011 ultra-wideband transceiver module. A network of two or more modules can be configured to wirelessly distribute trigger signals with a typical jitter of less than ±4 ns.

This approach can be applied in, e.g., structural health monitoring with ultrasonic guided waves. We used the solution to coordinate signal transmission and reception in a wireless sensor network.

## Graphical abstract

**Figure:**
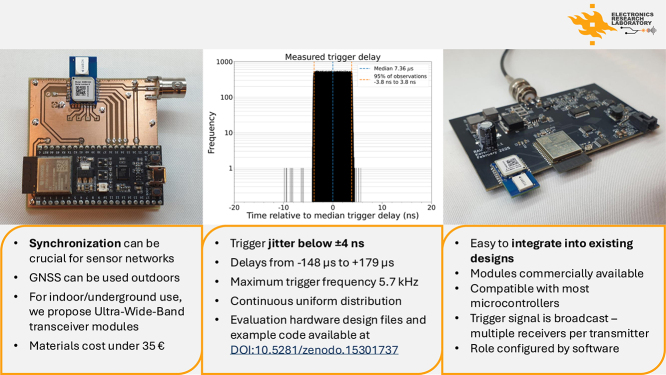

## Specifications table

**Table:** 

Hardware name	DWM1000-based trigger distribution system

Subject area	• Engineering and material science • General

Hardware type	• Field measurements and sensors

Closest commercial analog	Commercial GNSS modules can provide a synchronized signal that can be used for timing and triggering.

Open source license	The documentation and example source files are provided under the following licenses: • Hardware design files: CERN-OHL-P-2.0 • Software source code: MIT License • Documentation: CC-BY 4.0 creativecommons.org/licenses/by/4.0/

Cost of hardware	35 € per node, minimum of two nodes.

Source file repository	The source code is available on Zenodo DOI:10.5281/zenodo.15301737

## 1 Hardware in context

Distributed wireless sensor networks are used to record parameters from their surroundings. In many cases, like long-term monitoring, a timestamp is included to record the moment of the measurement. In some applications, millisecond accuracy can suffice — accomplished, e.g., by synchronizing real-time clocks over a wireless network. One example is the Network Time Protocol [1], which can be utilized in packet-switched networks, such as TCP/IP connections in a WiFi network.

Sensor networks that cover a wide geographic area and require an accuracy below one millisecond can utilize time signal transmitters like DCF77 [2] in central Europe or WWV [3] in North America. Distributed sensor networks with demanding synchronization requirements can further utilize GNSS signals (like GPS, GLONASS, BeiDou, Galileo). These signals are globally available and specialized receivers can derive a clock signal with an accuracy of several nanoseconds compared to the UTC time [4]. An example of a global sensing network that utilizes GNSS for precise timing information is Blitzortung.org [5], which monitors electromagnetic signals in the atmosphere to localize lightning strikes. As these waves travel at the speed of light, accurate timing information is required to narrow down the exact location of the strike.

However, GNSS signals are not available indoors or underground, and they can be jammed or spoofed, rendering equipment relying on the signals useless. Synchronization of wireless sensor nodes in a local area is also challenging in the field of non-destructive testing and structural health monitoring [6], [7].

To overcome this challenge, we present a wireless triggering system based on DWM1000 [8] IEEE 802.15.4-2011 ultra-wideband (UWB) transceiver modules utilizing the DW1000 IC (Qorvo Inc., Greensboro, North Carolina, USA) [9]. They are intended for wireless ranging with a resolution of 10 cm and communication at ranges of up to 300 m. The modules do not rely on external infrastructure, and they can be configured to generate an interrupt signal based on user-selectable events in the transmission and reception sequence.

This interrupt signal can then be utilized as a trigger signal with a jitter of less than ±4 ns. This mode of operation is not described by the manufacturer as it is not an intended use case for the modules. This article describes how the modules can be utilized as a wireless trigger distribution system.

We believe that this knowledge is beneficial for projects with long-range triggering requirements, such as the development of wireless sensor networks, which is why we present the following evaluation results as well as the hardware and code used for the experiments.

## 2 Hardware description

The trigger distribution system consists of two or more units with identical hardware. The hardware includes the DWM1000 UWB module, a host microcontroller (MCU) module and optional connectors which are soldered to an easy-to-manufacture two-layer printed circuit board. The host microcontroller is used to configure the UWB module and run a program loop for transmitting or receiving signals. Communication between the MCU and UWB modules is performed over the Serial Peripheral Interface (SPI).

The presented solution leverages the UWB module’s capability to output interrupt signals based on user-configured event masks. The interrupt is output to an interrupt request (IRQ) pin, which can be monitored by a host MCU. The pin can also be connected to components such as clocks or switches to enable peripheral circuits without intervention from the host MCU. This article is based on the DWM1000 ranging module, however similar modules or ICs with interrupt pins can conceivably be used in the same manner.

This study utilizes two DWM1000 transceivers, one operating as a transmitter (TX) and the other as a receiver (RX). However, the trigger distribution network may include more than one receiver, as the transmitted message is broadcast. Using more than one transmitter is possible, however additional software is required to ensure that transmission events do not overlap. The units can change their role (TX/RX) at any time as long as the necessary configuration change is implemented and managed by software.

In principle, any microcontroller capable of SPI communication and interrupt handling can act as the host. In this study, an ESP32-S2 (Espressif Systems, Shanghai, China) development board was used as the host microcontroller. Due to the relatively small footprint of the UWB module and its simple interface, integration with existing hardware is easy.

The synchronization capabilities of the presented system can be compared to GNSS-based solutions. The price range of GNSS modules extends both above and below the DWM1000, with midrange GNSS modules being cheaper.

The use cases for the two are different. GNSS modules excel in very large outdoor networks and continuous time-synchronization. Downsides include the reliance on existing infrastructure and the need of an unobstructed view of the sky.

The DWM1000 is better suited for cases where only one synchronization event is needed, e.g., to start data acquisition in a wireless sensor network with multiple receivers. Other advantages include the possibility of indoor and underground operation, as well as the ranging and communication capabilities intended by the manufacturer.

Line-of-sight between the transmitter and a receiver is recommended, but not strictly required, as using a reflected signal is possible [10], [11], [12], [13]. However, walls may prevent trigger distribution in situations where GNSS would work, such as outdoor areas with obstacles but a clear view of the sky.

The communication range of the DWM1000 can reach 300 m [14], which limits trigger distribution to a short range, in comparison with GNSS. The use of an external amplifier or a directional antenna [13] is possible, but local regulations may limit their use. Another option is to use some units as repeaters to extend the reach of the network.

Wired trigger distribution may be preferable in fixed setups over short distances, as cables are inexpensive and simple to use. However, cabling costs quickly add up and cheap, poorly shielded cables are susceptible to interference. The need for rerouting trigger cables may cause a setup to be less flexible than a wireless alternative.

In summary, the synchronization method presented here has strengths when compared to these alternatives.

• Wireless trigger signal with a jitter below ±4 ns
• Commercially available UWB module
• Usable with any SPI-capable host MCU
• Synchronization where GNSS is not reliable (indoor, underground)
• Transmitter and receiver roles selectable via software

## 3 Design files summary

A minimum working example to replicate this study is available on Zenodo (Table 1). The example includes a PCB layout and its schematic, as well as Arduino IDE compatible source code. The working principle and the register settings are discussed in Section 6. This allows the concept to transfer to any system that supports SPI communication and interrupt registration.

**Table 1:** Design files summary table.

Design filename	File type	Open source license	Location of the file

DWM1000Trigger _CAD.zip	PCB design files	CERN-OHL-P-2.0	Zenodo DOI:10.5281/zenodo.15301737
DWM1000Trigger _Code.zip	Source code	MIT License	Zenodo DOI:10.5281/zenodo.15301737

The PCB design file archive includes the schematic and PCB layout of the hardware for a single triggering unit. These files were created with KiCad version 8.09. The archive for the source code includes the necessary code to replicate the validation and characterization measurements detailed in this article.

## 4 Bill of materials

The bill of materials (Table 2) is for a single triggering unit as of April 2025. Two units are required to replicate this study, however more may be used. Designators marked with an asterisk are optional, and can be replaced with similar components (J1 to a BNC connector) or left unpopulated (TP1-5). The two-layer PCB presented in this article can be ordered from a PCB fabricator, but is designed to be easy to hand-etch. Thus, the cost of the PCB depends on the method of manufacture.

**Table 2:** Bill of materials (BOM) summary.

Designator	Component	Number	Cost per unit - currency	Total cost - currency	Source of materials	Material type
IC1	ESP32-S2-DEVKITM-1-N4R2	1	7.46 €	7.46 €	DigiKey	Semiconductor
IC2	DWM1000	1	21.79 €	21.79 €	DigiKey	Semiconductor
J1*	SMA edge connector	1	2.32 €	2.32 €	DigiKey	Other
J2, J3*	Pinheader, 1 × 1	2	0.09 €	0.18 €	DigiKey	Other
TP1-5*	Pinheader, 1 × 1	5	0.09 €	0.45 €	DigiKey	Other
–	PCB	1	–	–	–	Other

Total				32.20 €		

## 5 Build instructions

1. Assemble two units.
  - Do not supply power with the optional 3.3 V pinheader while the USB is connected.
2. Upload example code to both units via USB.
  - For the second unit, change “role” constant in example code from ‘T’ to ‘R’.
3. Connect oscilloscope probes to the trigger output.
  - Note that the grounds of both units may be connected via the probes depending on probe type.
4. Use the transmitting unit as the trigger source for the oscilloscope with a trigger level of 2 V.
5. Adjust oscilloscope delay to 7.4 µs with horizontal scale of 100 ns/div.
  - If the IRQ pin of either unit shows no activity, try resetting its host microcontroller or power cycling the unit in question.

**Fig. 1:**
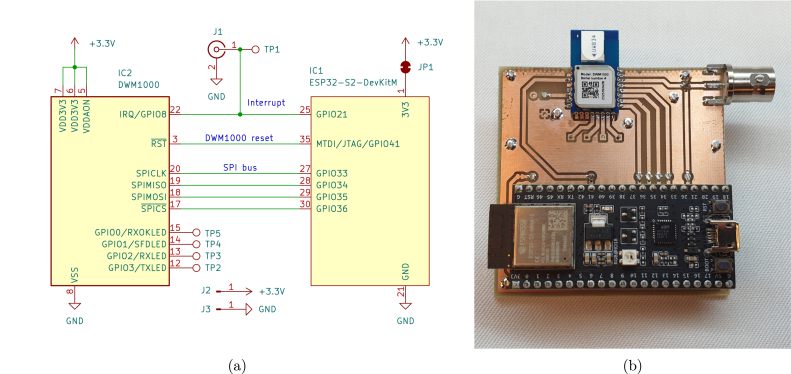
(a) Schematic of the minimum working example. The schematic includes the host MCU (IC1), the UWB module (IC2) and connectors. Either J1 or TP1 is required, whereas the rest of the connectors are optional. JP1 can be cut to separate the 3.3 V rails of IC1 and IC2. Pins not included in the schematic are left unconnected. (b) An assembled trigger unit. The host MCU (bottom) is connected to the UWB module (top). TP1 and J1 can be seen in the top-right corner of the 60 mm by 60 mm PCB. All nodes use the same hardware, as the TX & RX operation can be selected by software.

The minimum assembly consists of soldering the two modules (IC1, IC2), and either J1 or TP1 to the PCB. Other test points and connectors may be added to facilitate additional measurements. Additional pin sockets can be soldered under IC1 to easily reuse the host MCU. Fig. 1 shows the interconnection between the MCU and the UWB module. The assembled units can be powered by USB, or with the 3.3 V pin. The manufacturer of the ESP32 development board forbids using the 3.3 V pin when the USB is connected. A closed solder jumper (JP1) can be cut to separate the 3.3 V pins of the MCU and the UWB module.

Two units are required for a minimum working example. One of them operates as a transmitter (TX role) and broadcasts a message with the UWB module, while the other operates as a receiver (RX role). A network can consist of multiple receivers that all receive the same signal. TX and RX functionality is determined by software.

The source code for the minimum working example utilizing the ESP32 can be found at Zenodo DOI:10.5281/zenodo.15301737. The example is intended for use with the Arduino IDE [15] with the Arduino-ESP32 library [16] to enable easy testing. Version 2.3.4 of the Arduino IDE and version 3.1.1 of Arduino-ESP32 were used in this study.

The example code can be uploaded to the TX unit without changes. To program the RX unit(s), the “role” constant in the main source code file needs to be changed to ‘R’. Other optional settings include a custom transmission interval, and enabling of DWM1000 LED pins or serial prints.

## 6 Operation instructions

The default configuration of the example code can be used to replicate the validation presented in Section 7.1. The sample code was used with the “txinterval” constant set to zero. In this mode, the TX unit broadcasts a message as frequently as possible. At the start of each transmission, the IRQ pin is goes high until the System Event Status Register is cleared. The trigger signal appears as a logic-level pulse, with a predictable rise time and interval for the rising edge.

After initialization, the RX unit listens for a message. The IRQ pin goes high once the broadcast message is detected. The MCU then clears the interrupt event, reads the received message, and rearms the receiver for the next message. The trigger signal on the RX device appears after the TX unit’s trigger with a fixed delay, but is otherwise similar. Both RX and TX loops start automatically when the device is powered with no need for user intervention.

The UWB module allows different transmission and reception events to raise a flag, which can be masked to an interrupt. To change the event masks, such as in Section 7.2, the content of an instruction byte needs to be changed for the unit in question. In practice, one line of the source code needs to be commented and another uncommented. The premade event flag commands are labeled in the example code. Changing the event masks affects the median delay and probability distribution of the trigger signal.

The example code uses plain SPI commands with hard coded byte patterns for low memory requirements. The use of open source libraries for the UWB module (e.g., “arduino-dw1000” [17]) is possible in cases where memory is not a constraint.

For compatibility with any MCU, the configuration commands for the UWB modules are listed in Table 3 and Table 4. SPI communication with the UWB module is described in the documentation provided by the manufacturer [8, Sect. 1.3] [9, Sect. 2.2.1]. Only a minimal setup for the modules is presented here. Default settings that should be overridden for optimal operation are detailed in the user’s manual of the UWB module [9, Sect. 2.5.5].

**Table 3:** The write commands required to configure the UWB module for the TX role. These commands are transmitted to the UWB module via SPI. The start and end points of the example code loop are marked, as well as a place to extend the trigger interval.

Register address	Data	Notes	Manual section

0x0E	0x00000020	Include “Transmit Preamble Sent” as IRQ source	[9, Sect. 7.2.16]

Loop start			

0x09	0x54524947	Write message to TX buffer (example)	[9, Sect. 7.2.11]
0x0D	0x00000002	Start transmission	[9, Sect. 7.2.15]

Wait for desired trigger interval (optional)			

0x0F	0xFFFFFFFF	Clear all system status events	[9, Sect. 7.2.17]

Loop end

**Table 4:** The commands required to configure the UWB module for the RX role. For the read command, the number of bytes read should equal the length of the message programmed to the transmitter.

Register address	Data	Notes	Manual section

0x0E	0x00000200	Include “Receiver SFD Detected” as IRQ source	[9, Sect. 7.2.16]
0x04	0x20001200	Enable receiver-auto re-enable	[9, Sect. 7.2.6]
0x0D	0x00000100	Enable receiver	[9, Sect. 7.2.15]

Loop start			

Wait for IRQ to go high			

0x0F	0xFFFFFFFF	Clear all system status events	[9, Sect. 7.2.17]
0x11	Read 4 bytes	Read message (optional)	[9, Sect. 7.2.19]
0x0D	0x00000040	Disable receiver	[9, Sect. 7.2.15]
0x0D	0x00000100	Enable receiver	[9, Sect. 7.2.15]

Loop end			

### 6.1 Safety warnings

The presented example is a low-power circuit and is not, within reason, capable of causing damage. However, the following should be noted before adopting the system.

Before use, familiarize yourself with the DWM1000 user’s manual and follow the recommendations presented. Always follow local radio communication regulations and best practices.

In this example, no validation of the identity of the sender is done. Any received signal with a valid structure, per definition of the transmission protocol, will generate a trigger signal. Therefore, transmissions that do not belong to the trigger distribution network should be detected and handled.

One way to mitigate this is to add a unique message as payload. The receiver’s microcontrollers can validate this message before forwarding the trigger at the cost of additional software complexity and time delay. Additionally, the use of non-default transmission settings should be considered. The DWM1000 follows IEEE 802.15.4 UWB PHY, which includes settings such as center frequency, preamble codes, and pulse-repetition frequency of the preamble.

## 7 Validation and characterization

Validation was performed by using the example source code and measuring the produced trigger signals with an oscilloscope (HDO6104, Teledyne LeCroy, Chestnut Ridge, New York, USA). Trigger signals from both units were measured from a test point (TP1) with an oscilloscope probe (PP026, Teledyne LeCroy). In all experiments one unit was in the TX role and the other(s) in the RX role. All units were powered with the USB connector of the MCU development board (5 V, 0.5 A).

### 7.1 Trigger characterization

**Fig. 2:**
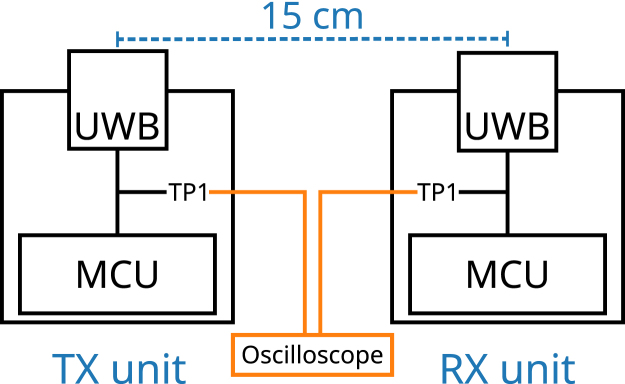
Schematic of the experimental setup. Test point 1 (TP1) of both units was monitored with an oscilloscope. The IRQ signal of the TX unit was used to trigger the oscilloscope, from which the delay to the RX unit’s IRQ signal was measured. The test points are shown in the schematic (Fig. 1).

To evaluate the reliability of the trigger signal, its delay and failure rate were measured. One TX unit and one RX unit were placed so that the UWB module antennas were parallel and 15 cm apart with clear line of sight (Fig. 2). Both antennas were raised 4.6 cm above a metal tabletop in a small indoor workspace. The “txinterval” constant was set to zero. Event masks “TXPRS” and “RXSFDD” were used for this experiment. One million trigger signal transmissions were evaluated.

**Fig. 3:**
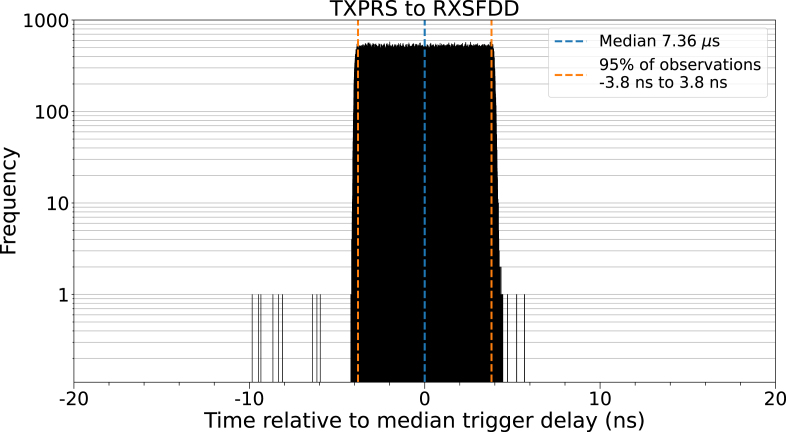
Measured trigger signal delays of one million trigger events. The distribution closely follows a continuous uniform distribution with 95 % of observations falling symmetrically within ±3.8 ns of the median delay (7.36 µs). 52 of the transmitted trigger events were missed by the receiver.

The mean trigger signal transmission interval was found to be 175.192 µs (≈5.7 kHz). The median delay for the received trigger signal was 7.360 µs, with 95 % of observations falling inside a 7.6 ns interval around the median. Of the transmitted signals, 52 were missed by the receiving unit (52 ppm). The histogram of the recorded delays (Fig. 3) shows a continuous uniform distribution.

The DWM1000’s system clock frequency (125 MHz) has a period of 8 ns. This period is close to the observed jitter of the trigger delay. Thus, we suspect that the jitter is caused by the processor of the module which updates the interrupt pin. The internal time resolution of the receiver is supposed to be below 1 ns as a precision of 10 cm is reported in the product brief [14].

### 7.2 Event mask combinations

To evaluate some of the other possible event mask combinations, the previous setup was used while the event mask was varied for both units. The event codes are those used in the user’s manual of the device [9, Sect. 7.2.16]. In all cases, only one of the event flags was enabled. Combining multiple flags, such as a flag for a successful event and its corresponding error flag (e.g., RXPHD and RXPHE), was not studied. Ten thousand transmitted trigger events were tested for each combination.

**Fig. 4:**
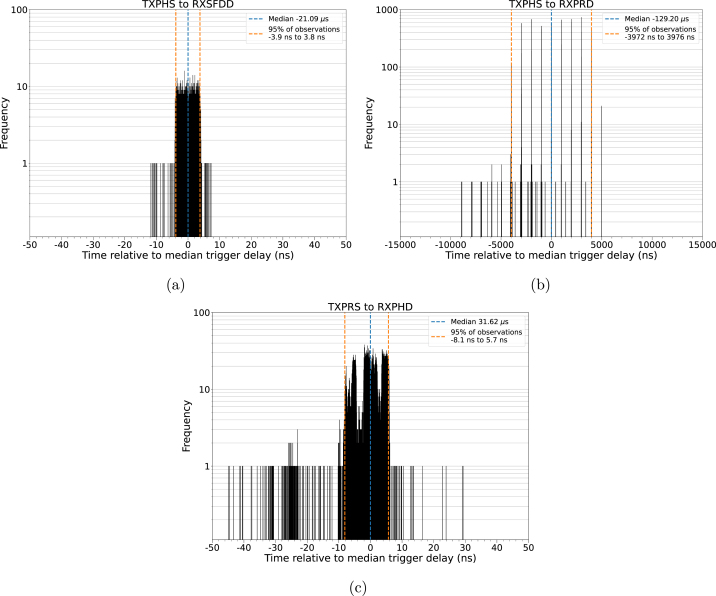
Sample distributions of event mask combinations. (a) Negative trigger delays are possible without impacting trigger quality. (b) Using RXPRD causes a discretized delay distribution and a thousandfold variance. (c) The probability distribution of most event flag combinations.

The measured median delays and their 95 % confidence intervals are shown in Table 5. Example distributions are presented in Fig. 4. Based on the results, TXFRB, TXPRS and TXPHS in combination with RXSFDD are recommended due to their continuous uniform distribution (Fig. 4(a)).

**Table 5:** Comparison of measured median trigger delays and 95 % confidence intervals for some possible event mask combinations. Positive values denote the IRQ pin of the receiving unit being raised high after the transmitter’s IRQ pin. RXSFDD produced the narrowest confidence intervals, whereas the use of RXPRD is not recommended due to the wide confidence intervals.

	TXFRB	TXPRS	TXPHS	TXFRS

RXPRD	26.4 µs, 7944 ns	-100.7 µs, 7944 ns	-129.2 µs, 7948 ns	-148.7 µs, 7948 ns
RXSFDD	134.5 µs, 9.6 ns	7.4 µs, 7.7 ns	-21.1 µs, 7.6 ns	-40.6 µs, 7.6 ns
RXPHD	158.8 µs, 13.2 ns	31.6 µs, 13.8 ns	3.2 µs, 13.8 ns	-16.3 µs, 14.2 ns
RXDFR	179.1 µs, 26.3 ns	51.9 µs, 12.5 ns	23.5 µs, 28.4 ns	4.0 µs, 14.4 ns

The use of the “receiver preamble detection” event (RXPRD) is not recommended due to the large 95 % confidence interval of the trigger jitter (>7.9 μs) and a discretized, comb-like distribution (Fig. 4(b)). This is most likely caused by the looping nature of the preamble.

The remaining combinations have a standard deviation comparable to the recommended ones, however their distributions are not uniform (Fig. 4(c)). These combinations can be used if the distribution pattern of the trigger delay is irrelevant, as the 95 % confidence interval for these combinations is narrow.

Notably, both positive and negative mean trigger delays can be achieved, such as the extreme examples of -148.4 µs and 179.1 µs. The smallest possible difference was 3.2 µs.

### 7.3 Multi-sensor networks

To evaluate the jitter in networks with multiple receivers, two receivers were placed 15 cm from a transmitter and the receiver-to-receiver delay of 250 000 trigger events was measured. Event masks “TXPRS” and “RXSFDD” were used for this experiment.

**Fig. 5:**
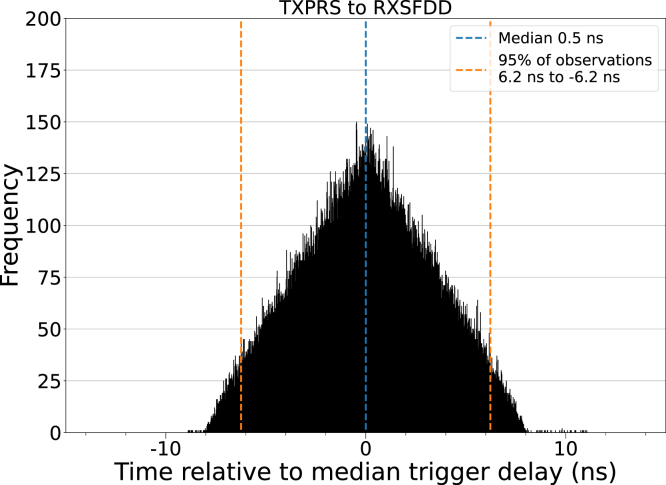
Measured trigger signal delays between two receivers. The symmetric triangular distribution suggests that the trigger events of each receiver are uncorrelated.

The resulting distribution is a symmetric triangle (Fig. 5), which is characteristic of the sum of two uncorrelated continuous uniform distributions with equal width. The median delay between receivers is near zero (0.5 ns) and 95 % of all observations fall within ±6.2 ns of the median. Based on this observation, it is likely that the jitter between two receivers is dominated by the aforementioned system clocks of each receiver. Due to the lack of correlation, the median delay between receivers is determined by their relative distances from the transmitter.

### 7.4 Delay and reliability at longer distances

The time delay Δt depends on the distance between the transmitter and the receiver Δx, as the signal propagates with the speed of light c ($\Delta t=\frac{\Delta x}{c}$). This also applies to transmitting trigger signals over wired connections, with potentially lower propagation speeds. Additional time delays caused by signal propagation in the circuit board and any downstream circuits should also be accounted for.

To evaluate the delay caused by signal propagation, a movable receiver (RX1) was used in conjunction with a stationary transmitter (TX) and a stationary receiver (RX2). The experiment was conducted in an office hallway with a width of 1.5 m and a height of 2.7 m. The transmitter was located at the centerline of the hallway and the stationary receiver was placed next to the transmitter at a distance of 25 cm. Event masks “TXPRS” and “RXSFDD” were used for this experiment. Ten thousand trigger events were recorded for each data point.

RX1 was initially placed 25 cm away from the transmitter at the center of the hallway. To calibrate the delay caused by a 30 m coaxial cable required for the measurement, the initial RX1 to RX2 delay at equal distance from the transmitter was measured with oscilloscope probes, after which RX1 was connected to the oscilloscope with the coaxial cable. The delay caused by the cable (146 ns) fits propagation velocity estimates found in the literature. Afterwards, the probability distribution of the delay between RX1 and RX2 was measured with RX1 at nine distances from 0.25 m to 25 m.

**Fig. 6:**
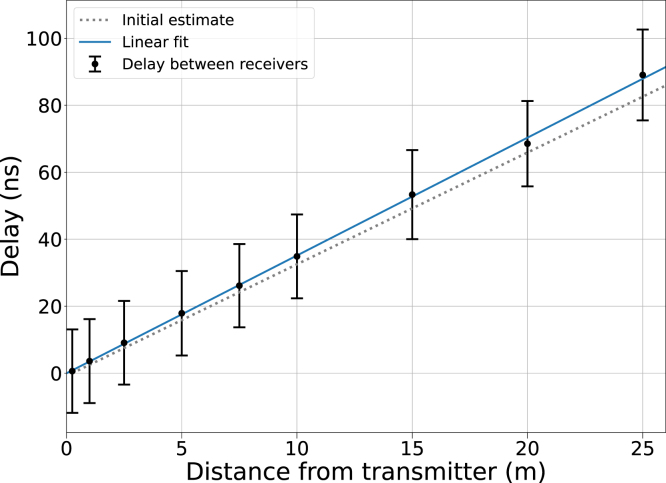
Measured trigger signal delays and 95 % intervals of the delay between two receivers. One receiver remained stationary and the other was moved relative to the transmitter. Based on a linear fit, the signal propagates at 95 % of the speed of light. The initial estimate using the speed of light in vacuum is shown with a dashed line. The jitter between receivers was constant at all distances, but 1.1 % of trigger events were missed at 25 m, compared to <0.5 % at all other distances. The 146 ns delay caused by a 30 m coaxial cable has been subtracted from all data points.

The delay was found to linearly increase with distance at a rate that suggests a signal propagation velocity of 95 % of the speed of light in vacuum (Fig. 6). The receiver-to-receiver jitter remained constant at all distances and the failure rate of the trigger, i.e., the fraction of cases where either receiver did not detect the signal, varied but stayed below 0.5 % up to 15 m. At 25 m, failure rate rose to 1.1 %, suggesting that non-default transmission settings may be required at longer distances.

### 7.5 Considering multipath effects

Multipath propagation of the signals affects the ranging capabilities of the DWM1000 and should be considered when the module is used for triggering. The effects depend on surrounding structures and how they reflect electromagnetic waves. The UWB module manufacturer provides extensive literature on the matter [10], [11], [12], [18].

If transmission takes place along the line of sight, this will account for the shortest trigger delay, which is directly proportional to the distance. But if the direct path is blocked, only diffracted or reflected signals can reach the receiving modules, extending the trigger delay by a fixed time. In the worst case, the signal strength of the direct path varies over time, causing the module to fluctuate between capturing the direct path and the reflected path, resulting in extended jitter.

The effect on practical applications will depend on the timing requirements and the environmental conditions of the installation. Therefore, no general recommendations on mitigating these effects can be given. Options might be to reposition the UWB modules to operate under line-of-sight conditions or to purposely block the weak direct path and enhance the reflected path using reflective structures. The use of directional antennas in multipath environments has also been studied [13].

## 8 Example use case

**Fig. 7:**
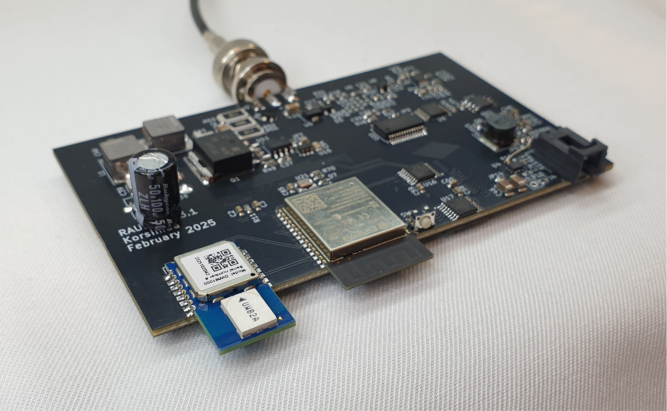
An ultrasound sensor unit with the UWB module installed. Wireless triggering enables multiple sensors to be used as a coordinated network for structural health monitoring. The UWB module is easy to integrate into existing designs due to its small footprint (<210 mm${}^{2}$).

The presented triggering scheme was used in a wireless sensor network (WSN) for ultrasound measurements in industrial environments [19]. The WSN consisted of four sensors (Fig. 7) with piezoelectric transducers coupled to a steel pipe. The sensors transmit and receive ultrasound that travels in the pipe wall. As the sensors are identical, they can be installed arbitrarily to balance the number of sensors and the need for sensing coverage. Due to the wireless nature of the network, it can be scaled up easily.

For this use case, the data from all sensor combinations was required, and thus all sensors needed to be interconnected. Sub-microsecond triggering was necessary for the analysis of the time-of-arrival of ultrasonic wave packets. The development of the sensor network was carried out indoors and indoor installation of the finished WSN was expected. Thus, GNSS could not be used.

The previous triggering scheme relied on a wired connection between the sensors. This solution fulfilled the timing requirements, but it was deemed difficult to scale up due to cost and cable routing concerns. Hundreds of meters of cable could be required for a network and the arbitrary placement of the sensors could be limited by wire routing capabilities in complex industrial environments.

Different wireless triggering systems were considered. Clock synchronization over WiFi was rejected due to the quickly arriving wave packets (100 µs). Continuous timing signals were not used due to the potentially complex software needed to coordinate the network. RF transceivers were found promising, as only a single synchronization pulse was required for each ultrasound transmission event. This single-event approach allows for the transceiver to be powered down when not in use to extend battery life. Eventually the presented trigger distribution system was developed, and the WSN met all of the above requirements.

## Device capabilities

• Typical trigger signal jitter (95 % confidence interval) less than ±4 ns
• Smallest trigger signal delay 3.2 µs
• Delays from -148 µs to 179 µs possible
• Shortest trigger interval 175 µs (5.7 kHz repetition frequency)
• Usable with any microcontroller that supports SPI communication and interrupts
• Trigger signal can be used to enable peripheral components (clocks, switches etc.)
• Normal UWB operation possible in combination with triggering
• The system can be used indoors, unlike GNSS
• Clear line of sight recommended between transmitter and receiver
• Triggering from reflected signals is possible, but dependent on surroundings
• Modules are used according to manufacturer specifications

## CRediT authorship contribution statement

**Julius Korsimaa:** Writing – review & editing, Writing – original draft, Visualization, Validation, Software, Methodology, Investigation, Formal analysis, Conceptualization. **Martin Weber:** Writing – review & editing, Writing – original draft, Supervision, Investigation, Funding acquisition, Conceptualization. **Edward Hæggström:** Writing – review & editing. **Ari Salmi:** Writing – review & editing, Supervision, Funding acquisition.

## Ethics statements

This research neither involved human subjects, animals nor the handling of personal data.

## Declaration of competing interest

The authors declare that they have no known competing financial interests or personal relationships that could have appeared to influence the work reported in this paper.
